# The role of cannabinoid CB1 receptors in the antinociceptive and reparative actions of mesenchymal stem cells in rats with peripheral neuropathic pain

**DOI:** 10.1002/ibra.12129

**Published:** 2023-08-24

**Authors:** Anna‐Maria V. Yerofeyeva, Sergey V. Pinchuk, Svetlana N. Rjabceva, Alla Y. Molchanova

**Affiliations:** ^1^ Institute of Physiology National Academy of Sciences of Belarus Minsk Belarus; ^2^ Institute of Biophysics and Cell Engineering National Academy of Sciences of Belarus Minsk Belarus

**Keywords:** anandamide, cannabinoid receptors, gait, mesenchymal stem cells, neuropathic pain

## Abstract

Mesenchymal stem cells (MSCs) can produce antinociceptive and reparative effects. Presumably, the MSCs‐induced antinociception may be partly due to the involvement of the endocannabinoid system. The study aimed to evaluate the antinociceptive and reparative effects of adipose‐derived MSCs (ADMSCs) upon pharmacological modulation of cannabinoid CB_1_ receptor in peripheral tissues or on ADMSCs' membranes in a rat model of peripheral neuropathy. ADMSCs were injected into the area of rat sciatic nerve injury (i) with no additional treatments, (ii) at the tissue CB_1_ receptor activation by endogenous agonist anandamide (AEA) or blockade with a selective AM251 antagonist; and (iii) preincubated with AEA or AM251. The evaluation of CB_1_ receptor activity involved analyzing nociceptive responses, gait parameters, and histology. Transplantation of ADMSCs upon activation of CB_1_ receptors, both on AMSCs' membranes or in the area of nerve injury, accelerated the analgesia and recovery of dynamic gait parameters, abolished static gait disturbances, and promoted the fastest nerve regeneration. Only blockade of CB_1_ receptors on ADMSCs shortened ADMSCs‐induced analgesia and decreased the number of preserved nerve fibers. CB_1_ receptors on ADMSCs significantly contribute to their pain‐relieving and tissue‐repairing capabilities by stimulating the growth factors secretion and suppressing the release of pro‐inflammatory cytokines. Peripheral CB_1_ receptors do not significantly influence ADMSC‐induced antinociception.

## Graphical abstract

The study aimed to evaluate the antinociceptive and reparative effects of adipose‐derived MSCs (ADMSCs) upon pharmacological modulation of cannabinoid CB_1_ receptor in peripheral tissues or on ADMSCs membranes in a rat model of peripheral neuropathy. The impact of the CB_1_ receptor state was evaluated by nociceptive responses, gait parameters, and histology. Obtained results indicate the predominant role of CB_1_ receptors on ADMSCs in their antinociceptive and reparative effects.

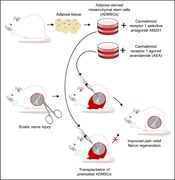

## 1 INTRODUCTION

Peripheral neuropathic pain (NP) is a group of chronic pain syndromes associated with damage or dysfunction in peripheral parts of the somatosensory nervous system.^1^ According to various estimates, NP covers 7%–20% of the adult population.^2^, ^3^ Currently used pharmacological methods for NP treatment are effective.^4^ However, these effects are relatively short and often are accompanied by significant side actions. Therefore, there is a need for new approaches to improve the life quality of this category of patients. Transplantation of mesenchymal stem cells (MSCs) is promising for the treatment of pain associated with peripheral nerve damage.^5^ The ability of MSCs to produce analgesia in both inflammatory and NP is proven in several studies,^6^, ^7^, ^8^, ^9^, ^10^, ^11^, ^12^, ^13^, ^14^, ^15^, ^16^, ^17^ but the mechanisms of these effects are still under investigation. As a component of the endocannabinoid system, cannabinoid receptor type 1 (CB_1_) is involved in the modulation of the nociceptive signal transduction, transmission, and processing both at the peripheral and central levels of the somatosensory nervous system.^18^ Cannabinoid receptors may impact the antinociceptive action of MSCs; the prerequisites for this have been demonstrated in recent studies in vitro.^16^, ^17^, ^18^ The mechanisms of MSCs' therapeutic effect can also be associated with their ability to secrete endogenous cannabinoids and their congeners, such as anandamide (AEA), 2‐arachidonylglycerol (2‐AG),^19^, ^20^ oleoylethanolamide (OEA), and palmitoylethanolamide (PEA).^19^ Pharmacological stimulation and blockade of CB_1_ receptors would help to understand their role in protective actions caused by MSCs.

This work aimed to study the effect of pharmacological modulation of CB_1_ receptors (on the membranes of adipose‐derived mesenchymal stem cells (ADMSCs) or in the soft tissues surrounding the nerve damage area) on nociceptive sensitivity, gait parameters, and changes in sciatic nerve histostructure.

## 2 MATERIALS AND METHODS

### 2.1 Animal grouping and treatment

A total of 60 male Wistar rats (8 to 10‐week‐old) were selected for the study based on adequate body weight and the absence of clinical signs of disease or injuries. The animals were kept in polypropylene cages in a temperature‐controlled room at 21–23°C and relative humidity of 30%–70%, a 12‐h light/dark cycle, and free access to food and water. Animal care and handling met the national and international requirements for the treatment of animals used for scientific purposes. The study protocol was approved by Bioethics Commission at the Institute of Physiology of the National Academy of Sciences of Belarus (protocol No. 1 dated February 2, 2022).

Rats were randomly assigned to the following groups, each consisting of 10 animals: rats with NP, rats with NP treated with adipose‐derived mesenchymal stem cells (ADMSCs) (NP+ADMSCs), NP rats administered with ADMSCs 5 min post i.m. injection AEA into the area of sciatic nerve injury (NP+ADMSCs+AEA), NP rats administered with ADMSCs 15 min post i.m. injection AM251, a potent CB_1_ receptor antagonist, into the area of sciatic nerve injury (NP+ADMSCs+AM251), NP rats administered with ADMSCs preincubated with AEA (NP+pre‐AEA‐ADMSCs), and NP rats received ADMSCs preincubated with AM251 (NP+pre‐AM251‐ADMSCs).

To induce NP, the animals were anesthetized with sodium pentobarbital (20 mg/kg, i.v.), and the sciatic nerve was sectioned proximal to its bifurcation into the tibial and the peroneal divisions with the removal of a 5‐mm segment to prevent regeneration. To prevent infection, the animals received ceftriaxone (100 mg/kg, s.c.) three consequential days after surgery.

### 2.2 ADMSCs isolating and transplantation

ADMSCs were isolated from the visceral adipose tissue of healthy rats. Fresh adipose tissue underwent treatment with a 0.25% collagenase solution and subsequent centrifugation (10 min at 400*g*). The resulting pellet was then resuspended in Dulbecco's modified eagle medium (DMEM) supplemented with 10% fetal calf serum, 2 mM l‐glutamine, and 1% standard antimycotic/antibiotic solution. The cells were seeded in Sarstedt culture flasks (Germany) and cultured in a CO_2_ incubator under standard conditions (5% CO_2_ at 37°C), with the growth medium being replaced every 72 h. ADMSCs from the third passage were utilized for this study. The flow cytometer FACSСanto II (Becton Dickinson) was employed to confirm the phenotype of the ADMSCs specifically by the presence of mesenchymal markers CD29, CD44, and CD90, and the absence of the hematopoietic marker CD45. The viable cell count was at least 90%. Before transplantation, ADMSCs were suspended in sterile phosphate‐saline buffer (PBS, pH = 7.2; Sigma‐Aldrich) at a final concentration of 1 × 10^6^ cells/mL. On the Day 7 post surgery, the cell suspension was injected around the sciatic nerve, with a dosage of 1 × 10^6^ cells/kg administered via four injections along the injury site perimeter.

### 2.3 Pharmacological modulation of CB_1_ receptors

The CB‐receptor agonist anandamide (AEA; Tocris Bioscience) was employed to activate CB_1_ receptors, whereas a specific CB_1_ receptor antagonist AM251 (Sigma‐Aldrich) was used as the blocker. AEA was dissolved in sterile saline to a final 100 µg/mL concentration. AM251 was dissolved in a mixture of sterile saline, Tween 80, and ethanol in a ratio of 18:1:1, to a final concentration of 1 mg/mL. AEA and AM251 were administered to the site of the sciatic nerve injury at dosages of 100 μg/kg and 100 μg per animal, respectively. To modulate CB_1_ receptors present on the membranes of ADMSCs before transplantation, the cells were pretreated for 24 h either with 5 μM AEA or with 10 μM AM251.

### 2.4 Nociceptive responses

Mechanical withdrawal threshold (MWT) was determined on days 0, 7, 14, 21, 28, 60, and 90 of the study in the Randall–Selitto test by the same named algesimeter (Panlab). Thermal withdrawal latency (TWL) was assessed on the same days in the Hot‐plate test with an appropriate algesimeter (Panlab) heated to 50 ± 0.1°C. Paw withdrawal, vocalization, hopping, or licking of the hind limbs were considered nociceptive responses. For each test mentioned, the measurements were repeated three times with an interval of 5–7 min.

### 2.5 Gait analysis

**Figure 1:**
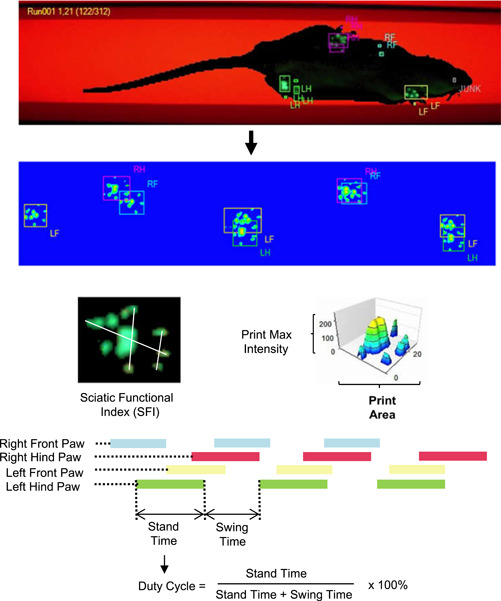
Characterization of studied pain‐related gait parameters in the CatWalk XT gait analysis system: dynamic gait parameters (stand time, swing time, duty cycle) and static gait parameters (print area; max intensity; sciatic functional index). [Color figure can be viewed at wileyonlinelibrary.com]

In parallel with the assessment of nociceptive sensitivity, detailed gait analysis was performed using the CatWalk XT hardware‐software complex version 10.6 (Noldus). Previously to the experiment, the animals adapted to the device without recording. Runnings were recorded in a dark ventilated room at a low noise level with a green‐light and red‐light intensities of 17.0 u, and a green light detection threshold of 13.0 u. For each rat, 3 runs with a similar run average speed, a maximum level of run maximum variation of up to 60%, and a run duration of up to 5.00 s had to be selected. Paw prints classification and value calculation of the gait parameters are made with supplied software. Key dynamic gait parameters were selected among others based on previous studies due to reflecting both the degree of tonic pain and the functional state of the sciatic nerve.^21^ The characteristics of the studied gait parameters are shown in Figure 1.

The gait parameters, except for the functional sciatic index (SFI), were calculated as a percentage of the contralateral hind limb. The calculation was used to eliminate the effect of running speed and animal body weight on gait parameters. SFI was calculated in software using a formula developed by Bain et al.^22^

### 2.6 Histological examination

On days 21 and 90 after surgery, the distal segment of the sciatic nerve was taken from previously euthanized animals (sodium thiopental, 200 mg/kg, i.v.) for histological examination. After removal, it was fixed in 10% neutral buffered formalin (Labiko). After dehydration, paraffin‐embedded tissue fragments were cut into 5 µm sections and stained with hematoxylin‐eosin according to the standard method. An Optec BK 5000 light microscope with a digital camera was used to view cross sections of the sciatic nerve at ×400 magnification. The percentage of damaged nerve fibers (NFs) and the number of Schwann cells (SCs) per 100 NFs were calculated.^23^ Calculation of these parameters was carried out in at least five fields of view.

### 2.7 Statistics

Statistical data processing was performed using Statistica version 10 (StatSoft Inc), and graphic processing was performed using OriginPro 2022 programs (OriginLab Corp). Data on nociceptive sensitivity and gait parameters are presented as M ± SE, where M is the mean value and SE is the standard error of the mean. Morphometric data are presented as Me [25%;75%], where Me is the median value and values of the lower and upper quartiles are given in square brackets. The data were tested for normal distribution using the Shapiro–Wilk test. Differences in nociceptive sensitivity and gait parameters were assessed using repeated measures analysis of variance (repeated‐measures ANOVA) followed by post hoc comparisons using the least significant difference method (LSD). Morphometric data were compared by the Kruskal–Wallis ANOVA test with subsequent post hoc comparisons using the multiple‐comparison *z* value test (Dunn's test). The conclusion about the statistical significance of differences was made at *p* < 0.05.

## 3 RESULTS

### 3.1 Nociceptive responses to mechanical and thermal stimuli

**Figure 2:**
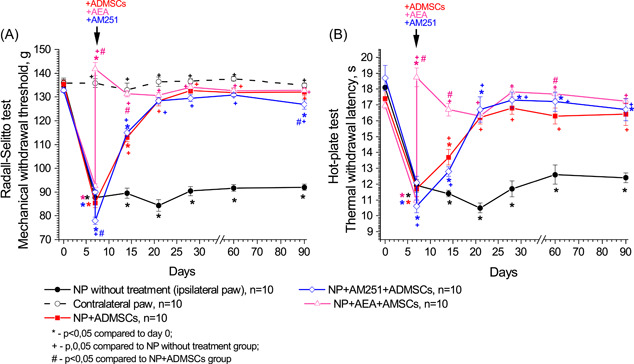
Impact of pharmacological modulation of CB_1_ receptors in the soft tissues surrounding the site of sciatic nerve injury on nociceptive responses to mechanical (A) and thermal (B) stimuli in rats with neuropathic pain (NP). The arrow indicates the time of transplantation of ADMSCs. Data were assessed by repeated measures ANOVA with post hoc comparisons using the lowest significant differences method. [Color figure can be viewed at wileyonlinelibrary.com]

**Figure 3:**
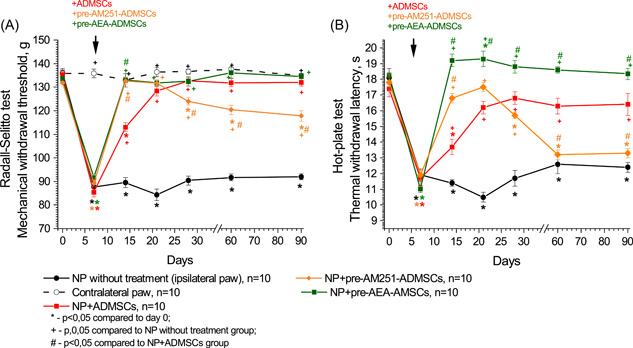
Impact of pharmacological modulation of CB_1_ receptors on membranes of adipose‐derived mesenchymal stem cells (ADMSCs) on nociceptive responses to mechanical (A) and thermal (B) stimuli in rats with neuropathic pain. The arrow indicates the time of transplantation of ADMSCs. Data were assessed by repeated measures ANOVA with post hoc comparisons using the lowest significant differences method. [Color figure can be viewed at wileyonlinelibrary.com]

On the seventh day of post‐NP modeling, the sensitivity to mechanical and thermal stimuli increased. This was confirmed by a decrease in the MWT of the affected limb by 35.5% (from 136.0 ± 1.9 g to 87.7 ± 2.0 g, Figures 2A and 3A), and a reduction in TWL by 34.3% (from 18.1 ± 0.6 s to 11.9 ± 0.4 s, *p* < 0.001 compared to the values on Day 0, Figures 2B and 3B). Over the course of the study, there was no indication of the MWT and TWL returning to their initial levels. The MWT of the healthy limb on the opposite side did not demonstrate any significant changes throughout the study (*p* > 0.05 compared to Day 0, Figures 2A and 3A).

Following a single intramuscular injection of ADMSCs into the site of sciatic nerve injury, there was a notable MWT improvement of the affected limb by 32.3% on Day 14 after surgery (from 85.4 ± 2.0 g to 113.0 ± 1.9 g, *p* < 0.001 compared to the Day 7, Figures 2A and 3A). Additionally, the TWL exhibited a significant increase of 17.1% (from 11.7 ± 0.5 s to 13.7 ± 0.5 s, *p* < 0.001 compared to the values on Day 7, Figures 2B and 3B). By the 21st day after surgery, the MWT and TWL had fully recovered to their initial levels. From Day 90 onward, the parameters of nociceptive responses demonstrated no significant differences compared to the baseline values, and they were significantly higher than those observed in NP cases without treatment (*F*(6,90) = 56,79, *p* < 0.001).

Intramuscular injection of AEA into the area of sciatic nerve damage after 5 min led to a sharp increase in the MWT of the ipsilateral limb by 53.1% (from 92.6 ± 0.9 g to 141.7 ± 2.7 g, *p* < 0.001 compared to Day 7, Figure 2A) and TWL by 70.0% (from 11.0 ± 0.2 s to 18.7 ± 0.6 s, *p* < 0.001 compared to Day 7, Figure 3B). Subsequent transplantation of ADMSCs led to a higher increase in MWT by Day 14 of the study compared to the NP+ADMSCs group (up to 131.3 ± 1.3 g; *p* < 0.001) and TWL (up to 16.7 ± 0.4 s; *p* < 0.001). These parameters did not significantly differ compared to Day 0, but significantly differ compared to the NP+ADMSCs group (*F*(6,84) = 10,598, *p* < 0.001 and *F*(6,84) = 5,115, *p* < 0.001, respectively), and untreated NP group (*F*(6,90) = 75,920, *p* < 0.001 and *F*(6,90) = 41,237, *p* < 0.001, respectively).

Intramuscular injection of AM251 into the area of sciatic nerve injury did not affect nociceptive sensitivity (Figure 2A,B). After transplantation of ADMSCs, 15 min after administration of AM251, the dynamics of changes in nociceptive responses to mechanical and thermal stimuli were identical to the NP+ADMSCs group (*F*(6,84) = 1,417, *p* > 0.05). A significant decrease in the MWT of the ipsilateral limb was noted on Day 90 after surgery (up to 126.8 ± 2.0 g, *p* < 0.05 compared to the Day 0 values, Figure 2A).

The transplantation of pre‐AEA‐ADMSCs into the site of sciatic nerve injury resulted on the 14th day in a notably higher MWT increase compared to the NP+ADMSCs group (*F*(6,84) = 8,130, *p* < 0.001), reaching up to 133.1 ± 1.2 g, *p* < 0.001 (Figure 3A), as well as an elevated TWL (*F*(6,84) = 8075, *p* < 0.001), reaching up to 19.2 ± 0.4 s, *p* < 0.001 (Figure 3B). Importantly, these parameters did not display any significant differences compared to the values on Day 0. Furthermore, the MWT did not significantly differ from the values on Day 0 till the end of the study. On Day 21 after surgery, there was an increase in TWL (reaching up to 19.3 ± 0.5 s, *p* < 0.04 compared to Day 0, Figure 3B).

The administration of pre‐AM251‐ADMSCs resulted in the recovery of MWT to the initial values by the 14th day after surgery (up to 132.7 ± 1.6 g, *F*(6,84) = 57,048 *p* < 0.001 compared to NP without treatment and *F*(6,78) = 20,597, *p* < 0.001 compared to NP+ADMSCs group, Figure 3A). Similarly, the TWL returned to the initial values on the 14th day after surgery (up to 17.0 ± 0.3 s, *F*(6,84) = 31,395 *p* < 0.001 compared to NP without treatment and *F*(6,78) = 14,502, *p* < 0.001 compared to NP+ADMSCs group, Figure 3B). From the 28th day after surgery onward, there was a noticeable decline in the MWT compared to the NP+ADMSCs group and the values on Day 0 (*p* < 0.05). Additionally, by the 60th day, a reduction in TWL was observed compared to untreated NP rats (*p* < 0.05). The trend toward a decrease in both MWT and TWL persisted until Day 90 (Figure 3A,B).

#### 3.1.1 Gait parameters

**Figure 4:**
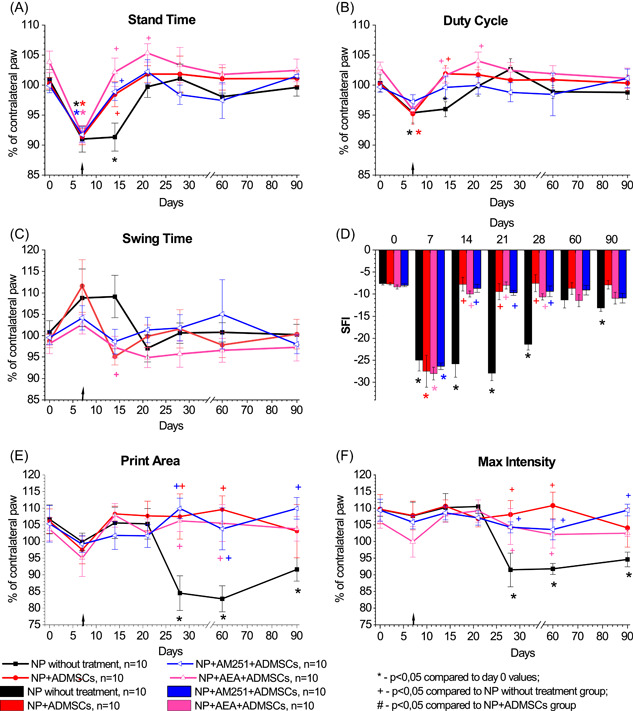
Impact of pharmacological modulation of CB_1_ receptors of the area of sciatic nerve injury on gait parameters (A–F) in rats with neuropathic pain. The arrow indicates the time of transplantation of ADMSCs. Data were compared by repeated measures ANOVA with post hoc comparisons using the lowest significant differences method. [Color figure can be viewed at wileyonlinelibrary.com]

**Figure 5:**
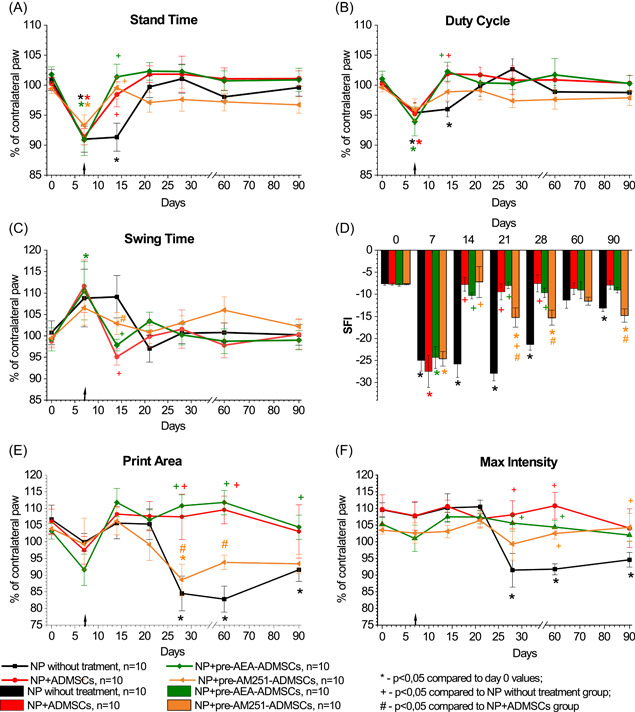
Impact of pharmacological modulation of CB_1_ receptors on membranes of adipose‐derived mesenchymal stem cells (ADMSCs) by pretreatment on changes in gait parameters (A–F) in rats with neuropathic pain. The arrow indicates the time of transplantation of ADMSCs. Data were analyzed using repeated measures ANOVA with post hoc comparisons with the lowest significant differences method. [Color figure can be viewed at wileyonlinelibrary.com]

In the group of NP without treatment, there were temporary disruptions in the dynamic gait parameters. Significant changes were observed in stand time (*F*(6,78) = 2483, *p* < 0.03) and duty cycle (*F*(6,78) = 2548, *p* < 0.03). On the seventh day following the surgery, the duration of the ipsilateral limb's stand time decreased to 91.0% (*p* < 0.001, Figures 4A and 5A), and the duty cycle decreased to 95.4% (*p* < 0.001, Figures 4B and 5B). No significant differences in swing time were observed throughout the study (*F*(6,78) = 0736, *p* > 0.05, Figures 4C and 5C).

SFI in the untreated NP group showed a significant decrease (*F*(6,78) = 11,346, *p* < 0.001) of 2.3 times on Day 7 after surgery (from −7.65 ± 0.30 to −24.99 ± 2.45, *p* < 0.001 compared to Day 0, Figures 4D and 5D). On Day 90, the SFI in this group was −13.13 ± 0.79, indicating a lack of recovery to the initial level (*p* < 0.01, Figures 4D and 5D).

Static gait parameters, specifically print area, and maximum intensity, were impaired in rats with untreated NP starting from Day 28 after surgery (*F*(6,78) = 2818, *p* > 0.02 and *F*(6,78) = 4190, *p* > 0.002, respectively, Figures 4E,F and 5E,F). The print area significantly decreased to 84.5% of the contralateral limb (*p* < 0.001 compared to Day 0, Figures 4E and 5E). The maximum intensity also significantly decreased to 91.5% of the contralateral limb (*p* < 0.001 compared to Day 0, Figures 4F and 5F). By Day 90, there was no observable tendency to restore the static gait parameters.

The transplantation of ADMSCs into the area of sciatic nerve injury resulted in the accelerated recovery of dynamic gait parameters such as stand time (*F*(6,90) = 7087, *p* < 0.01), duty cycle (*F*(6,90) = 3380, *p* < 0.01) and SFI (*F*(6,78) = 13,528, *p* < 0.001) compared to untreated rats. By Day 14 after surgery, the stand time significantly increased to 98.4% (*p* < 0.05 compared to the untreated NP group, *p* > 0.05 compared to Day 0, Figures 4A and 5A). The duty cycle also increased by Day 14 after surgery to 101.9% (*p* < 0.005 compared to the untreated NP group, *p* > 0.05 compared to Day 0, Figures 4B and 5B). Additionally, the SFI showed a recovery by Day 14 after surgery, reaching −7.75 ± 1.57 (*p* < 0.001 compared to the untreated NP group, *p*> 0.05 compared to Day 0, Figures 4D and 5D). No further statistically significant changes were observed in these parameters compared to Day 0 (Figures 4D and 5D). There were no significant changes in print area (Figures 4E and 5E) or maximum intensity (Figures 4F and 5F) observed in this group. However, compared to the untreated NP group, an increase in print area and maximum intensity was observed (*F*(6,90) = 3511, *p* < 0.001 and (*F*(6,90) = 3627, *p* < 0.003, respectively). Significant changes between these groups were noted on Day 28 after surgery (*p* < 0.005) and on Day 60 after surgery (*p* < 0.02).

Both AEA and AM251 injection into the area of sciatic nerve injury followed by transplantation of ADMSCs led to the recovery of stand time (Figure 4A), duty cycle (Figure 4B), and SFI (Figure 4D) by Day 14 after surgery. In addition, in the NP+AEA+ADMSCs and NP+AM251+ADMSCs groups, there were no disturbances in the print area and max intensity (Figure 4E,F). No significant differences were found in all studied gait parameters in the NP+AEA+ADMSCs and the NP+AM251+ADMSCs groups compared to the NP+ADMSCs group.

The transplantation of pre‐AEA‐ADMSCs in rats with NP resulted in the restoration of stand time, duty cycle, and SFI by Day 14 after surgery (Figure 5A,B,D). Additionally, the transplantation effectively eliminated the disruptions in static gait parameters (Figure 5E,F). No statistically significant differences were observed in the analyzed gait parameters when compared to the NP+ADMSCs group.

At the same time, after transplantation of pre‐AM251‐ADMSCs, recovery of the stand time and duty cycle parameters was also observed by Day 14 after surgery (Figure 5A,B). No significant differences were found compared to the NP+ADMSCs group (*F*(6,78) = 1360, *p* > 0.05 and *F*(6,78) = 0497, *p* > 0.05, respectively). Administration of pre‐AM251‐ADMSCs also led to a short‐term recovery of SFI by Day 14 after surgery (*F*(6,78) = 6,781, *p* < 0.0001 compared to the untreated NP group, Figure 5D). However, from Day 21, this group showed a downward trend in SFI (*p* < 0.02 compared to the Day 0 values; *p* < 0.001 compared to NP without treatment group, *p* < 0.005 compared to NP+ADMSCs group). From Day 28 onward, the SFI index did not differ significantly from NP without treatment group. After the transplantation of pre‐AM251‐ADMSCs, disturbances in static gait parameters were evident. There was a decrease in a print area compared with the NP+ADMSCs group (*F*(6,78) = 2223, *p* < 0.05) on Day 28 (*p* < 0.02) and Day 60 after surgery (*p* < 0.05, Figure 5E). At the same time, this parameter did not differ significantly from the untreated NP group (*F*(6,84) = 0795, *p* > 0.05). By Day 90, the print area did not differ significantly from untreated NP and NP+ADMSCs groups. Regarding max intensity, despite observing a downward trend from 28 days after surgery, no significant changes were found in comparison with the NP+ADMSCs group during the study (*F*(6,78) = 0433, *p* > 0.05, Figure 5F).

#### 3.1.2 Morphological analysis of sciatic nerve structure

**Figure 6:**
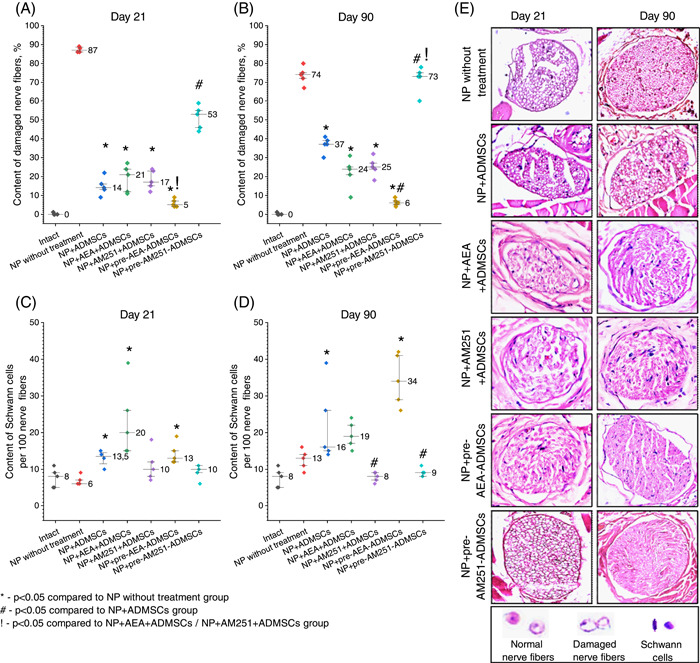
Impact of pharmacological modulation of CB_1_ receptors on the counts of damaged nerve fibers (A, B) and Schwann cells (SCs) (C, D) of the distal segment of the sciatic nerve in rats with neuropathic pain (NP) (E) Histostructure of the distal segment of the sciatic nerve of experimental rats with NP, stained with hematoxylin and eosin, ×400 magnification. Data were analyzed using Kruskal–Wallis ANOVA with post hoc comparisons (Dunn's test). [Color figure can be viewed at wileyonlinelibrary.com]

Figure 6 illustrates the morphometric analysis results of damaged nerve fibers (NFs) and Schwann cells (SCs) in the distal segment of the sciatic nerve. The assessment of NFs' condition was based on myelin sheath integrity and the position of the axial cylinder within the fibers. In normal NFs, distinct axial cylinders were observed, surrounded by a uniformly stained myelin sheath with clear boundaries. Whereas, damaged NFs exhibited swelling, vacuolic degeneration of the myelin sheath, and indistinct boundaries of the nerve fiber. In some cases, the axial cylinder was displaced toward the periphery or was undetectable in the histological sections (Figure 6E).

Significant differences between groups were found when comparing the number of damaged NFs both on Day 21 (*H*(5) = 24,048, *p* < 0.001) and Day 90 (*H*(5) = 26,256, *p* < 0.001), as well as the number of SCs (*H*(5) = 20,355, *p* = 0.011 and *H*(5) = 24,895, *p* < 0.001, respectively). Throughout the study, the untreated NP group exhibited elevated levels of damaged NFs (Figure 6A,B). However, on Day 21 post‐ADMSCs injection, both a reduction in the number of damaged NFs and an increase in SCs compared to the untreated animals were observed (*p* = 0.002 and *p* = 0.040, respectively, Figure 6A,C). This group maintained a low number of dystrophic altered NFs on Day 90 (*p* = 0.001, Figure 6B).

In comparison to the NP+ADMSCs group, the administration of pre‐AM251‐ADMSCs resulted in an increase in the content of damaged NFs both on Days 21 (*p* = 0.007, Figure 6A) and Day 90 (*p* = 0.029, Figure 6B). Conversely, a significant decrease in the number of damaged NFs was observed in the NP+pre‐AEA‐ADMSCs group on Day 90 after surgery (*p* = 0.002, Figure 6B). By the same time point, there was also a noteworthy decrease in the number of SCs in the NP+AM251+ADMSCs group (*p* = 0.007) and the NP+pre‐AEA‐ADMSCs group (*p* = 0.002, Figure 6D). The NP+pre‐AEA‐ADMSCs group exhibited the highest number of SCs per 100 NFs (*p* = 0.006) compared to the untreated NP, but no significant differences were found when compared to the NP+ADMSCs group (*p* = 0.335).

## 4 DISCUSSION

MSCs have a long‐term analgesic effect in peripheral NP, as demonstrated in several studies.^7^, ^8^, ^9^, ^10^, ^11^, ^12^, ^13^, ^14^, ^15^, ^16^, ^17^ Cannabinoid CB_1_ receptors are involved in the modulation of nociceptive signal transduction and transmission both in peripheral tissues and in the central nervous system and serve as one of the targets for pain relief in NP.^18^, ^24^ CB_1_ receptors are also involved in axonal regeneration after injury.^25^ At the same time, CB_1_ receptors are expressed on ADMSCs,^26^ and they are involved in maintaining cell viability and metabolic activity.^27^ CB_1_ receptors also direct toward the differentiation of MSCs in the bone marrow and adipose tissue.^28^ In this regard, the authors suggested that CB_1_ receptors may be one of the mediators of the antinociceptive and reparative action of ADMSCs.

In this study, the analgesic effects of ADMSCs were evident through the alleviation of mechanical and thermal hyperalgesia (Figures 2 and 3) AND the restoration of gait disturbances in rats following sciatic nerve axotomy (Figures 4 and 5). Interestingly, the blocking of CB_1_ receptors in the area of the sciatic nerve injury did not impact the actions of ADMSCs. However, the activation of CB_1_ receptors in the same area by AEA before ADMSC transplantation resulted in a faster reduction of nociceptive responses compared to the administration of ADMSCs alone (Figure 3), although it did not affect the rate of recovery of gait parameters (Figure 4). This effect can be attributed to the fact that AEA, as an endogenous cannabinoid, exerts its effects through various subtypes of cannabinoid receptors (such as CB_2_ receptors), as well as nonclassical cannabinoid receptors (GPR55, GPR18), PPARα, and the vanilloid receptor TRPV1.^29^, ^30^ Based on these findings, we propose that the analgesic effect of ADMSCs is unlikely to be mediated by the activation of peripheral CB_1_ receptors but may involve other cannabinoid receptor subtypes, such as CB_2_ receptors. Particularly, the involvement of CB_2_ receptors in the immunosuppressive properties of MSCs has been shown in several studies.^19^, ^27^, ^31^ CB_2_ receptors on microglia have been shown to modulate the course of NP.^32^, ^33^ Besides, recent studies highlighted the implementation of CB_2_ receptors presented on peripheral tissues in the alleviation of NP.^34^, ^35^ In this study, we are focused on investigating CB_1_ receptors, due to the lack of such studies, so its role seemed to be underestimated.

The administration of ADMSCs pretreated with AEA resulted in a faster recovery of nociceptive responses compared to the injection of ADMSCs alone (Figure 3). Moreover, the pre‐AEA‐ADMSCs showed sustained attenuation of nociceptive responses to thermal stimuli during the later stages of the study. Notably, the blockade of CB_1_ receptors on ADMSCs significantly reduced their antinociceptive action. However, the analysis of gait parameters did not reveal an enhanced effect of ADMSCs under the influence of AEA (Figure 5). On the other hand, the administration of pre‐AM251‐ADMSCs resulted in disturbances in SFI and static gait parameters, similar to those observed in the untreated NP group. These findings suggest that the activation of CB_1_ receptors on ADMSCs is necessary for the development of their antinociceptive effect upon local administration.

The histological examination of the distal segment of the sciatic nerve revealed the reparative effect of ADMSCs, characterized by the inhibition of degenerative changes in NFs and the stimulation of SCs proliferation (Figure 6). Interestingly, the stimulation of peripheral CB_1_ receptors did not have a significant impact on the reparative effect of ADMSCs. However, the blockade of peripheral CB_1_ receptors before ADMSC administration abolished the stimulation of SCs proliferation but did not affect the number of damaged NFs. In contrast, the transplantation of pre‐AEA‐ADMSCs resulted in a more robust recovery of NFs compared to the injection of ADMSCs alone and also promoted increased proliferation of SCs during the later stages of the study. Conversely, the transplantation of pre‐AM251‐ADMSCs had the opposite effect. The morphometric data obtained are consistent with the results of the tests assessing nociceptive reactions and suggest the predominant role of CB_1_ receptors on the membranes of ADMSCs in the development of their reparative effect.

The increased secretion of endogenous cannabinoids in the area of nerve damage may contribute to the therapeutic effect of locally administered ADMSCs. Previous research conducted by Ruhl and colleagues demonstrated that the activation of CB_1_ receptors on ADMSCs stimulates the secretion of various factors, including vascular endothelial growth factor (VEGF), transforming growth factor‐beta (TGF‐β), and hepatocyte growth factor (HGF).^27^, ^36^ Moreover, when the culture of MSCs was exposed to a lipopolysaccharide‐induced pro‐inflammatory environment, the addition of AEA suppressed pro‐inflammatory cytokine secretion.^19^, ^37^ Therefore, the prestimulation of CB_1_ receptors on ADMSCs can enhance their therapeutic properties, as the current study did demonstrate. It is worth noting that, to our knowledge, no other studies have been found that investigate the effects of ADMSCs in combination with pharmacological modulation of CB_1_ receptors in an in vivo experiment.

## 5 CONCLUSION

Obtained data suggest that antinociceptive and reparative effects of ADMSCs injected into the site of peripheral nerve damage are more likely linked to the activation of the stem cell's CB_1_ receptors. The ability of ADMSCs to alleviate mechanical allodynia and thermal hyperalgesia in conditions of NP is not significantly influenced by peripheral CB_1_ receptors. The CB_1_ receptor's impact on ADMSCs actions is associated with stimulating the growth factors secretion and suppressing the release of pro‐inflammatory cytokines. Further research is necessary to gain a deeper understanding of how CB_1_ receptors are involved in the therapeutic properties of ADMSCs.

## AUTHOR CONTRIBUTIONS

Anna‐Maria V. Yerofeyeva conducted data acquisition as well as data analysis and also drafted the manuscript. Sergey V. Pinchuk conducted stem cell preparation and in vitro data collection. Svetlana N. Rjabceva analyzed the histology data and revised the manuscript. Alla Y. Molchanova made the conception and design of the study and revised the manuscript. All authors approved the final version of the manuscript.

## CONFLICT OF INTEREST STATEMENT

The authors declare no conflict of interest.

## TRANSPARENCY STATEMENT

The authors affirm that this manuscript is an honest, accurate, and transparent account of the study being reported; that no important aspects of the study have been omitted; and that any discrepancies from the study as planned (and, if relevant, registered) have been explained.

## ETHICS STATEMENT

The animal experimental design in this study was approved by Bioethics Commission at the Institute of Physiology of the National Academy of Sciences of Belarus (protocol No. 1 dated February 2, 2022). Animal care and handling met the national and international requirements for the treatment of animals used for scientific purposes.

## Data Availability

Data within this article is available and can be obtained on request.
